# Competition for land

**DOI:** 10.1098/rstb.2010.0127

**Published:** 2010-09-27

**Authors:** Pete Smith, Peter J. Gregory, Detlef van Vuuren, Michael Obersteiner, Petr Havlík, Mark Rounsevell, Jeremy Woods, Elke Stehfest, Jessica Bellarby

**Affiliations:** 1 Institute of Biological and Environmental Sciences, University of Aberdeen, 23 St Machar Drive, Aberdeen AB24 3UU, UK; 2 Scottish Crops Research Institute (SCRI), Invergowrie, Dundee DD2 5DA, UK; 3 Netherlands Environmental Assessment Agency (PBL), PO Box 303, 3720 Bilthoven, The Netherlands; 4 International Institute for Applied Systems Analysis (IIASA), Schlossplatz 1, 2361 Laxenburg, Austria; 5 Centre for the Study of Environmental Change and Sustainability (CECS), School of Geosciences, University of Edinburgh, Drummond Street, Edinburgh EH8 9XP, UK; 6 Centre for Environmental Policy, Faculty of Natural Sciences, Imperial College London, South Kensington, London SW7 2AZ, UK

**Keywords:** competition for land, land use, agriculture, forestry, policy

## Abstract

A key challenge for humanity is how a future global population of 9 billion can all be fed healthily and sustainably. Here, we review how competition for land is influenced by other drivers and pressures, examine land-use change over the past 20 years and consider future changes over the next 40 years.

Competition for land, in itself, is not a driver affecting food and farming in the future, but is an emergent property of other drivers and pressures. Modelling studies suggest that future policy decisions in the agriculture, forestry, energy and conservation sectors could have profound effects, with different demands for land to supply multiple ecosystem services usually intensifying competition for land in the future.

In addition to policies addressing agriculture and food production, further policies addressing the primary drivers of competition for land (population growth, dietary preference, protected areas, forest policy) could have significant impacts in reducing competition for land. Technologies for increasing per-area productivity of agricultural land will also be necessary. Key uncertainties in our projections of competition for land in the future relate predominantly to uncertainties in the drivers and pressures within the scenarios, in the models and data used in the projections and in the policy interventions assumed to affect the drivers and pressures in the future.

## 1. Introduction

The UK Foresight Global Food and Farming Futures Project is considering how a future global population of 9 billion can all be fed healthily and sustainably (Foresight 2009). The project has identified 19 ‘drivers’ (with subcategories) affecting food and farming in the future, one of which is competition for land. The purpose of this review is to examine competition for land, and to consider the direct and indirect pressures and drivers affecting it. The scope of the review is global and the time scale considered is the past 20 years and the next 40 years (1990–2050).

In addition to agriculture, use is included for forestry, non-food crops and protected areas for biodiversity, as well as use of land for bioenergy and land degradation/restoration. The impact of policy on influencing each of these factors is discussed in §2*c*.

We summarize the quantitative information on changes in land use and land quality over the last 20 years, both globally and disaggregated according to the major regions of the world. The most recent synthesis of agricultural land-use change was conducted for the International Assessment of Agricultural Knowledge, Science and Technology for Development (IAASTD), particularly the chapter ‘Outlook on agricultural changes and its drivers’, dealing with land-use and land-cover change (van Vuuren *et al*. 2008). That study collated projections from the *Land use and cover change synthesis* book (Alcamo *et al*. 2005), the scenarios from the Global Scenarios Group (Raskin *et al*. 2002), IPCC Special Report on Emissions Scenarios (SRES) (IPCC 2000), the Millennium Ecosystem Assessment (MEA 2005), UNEP's Global Environment Outlook (UNEP 2002) and some models from the EMF-21 study of the Energy Modelling Forum (e.g. Kurosawa 2006; van Vuuren *et al*. 2006). We expand on that synthesis by adding more recent studies in §§4 and 5. In these sections, we present projections of land-use change to 2050 and examine the impact of changes in non-agricultural policy (e.g. forest and protected land policy) on competition for land. We briefly examine the assumptions upon which the projections are based and identify the main areas of uncertainty.

We conclude by assessing and ranking the most important external factors that may affect the land available for agriculture between now and 2050, and by discussing future needs to reduce uncertainties in these projections.

## 2. Factors affecting competition for land

**Figure 1.**
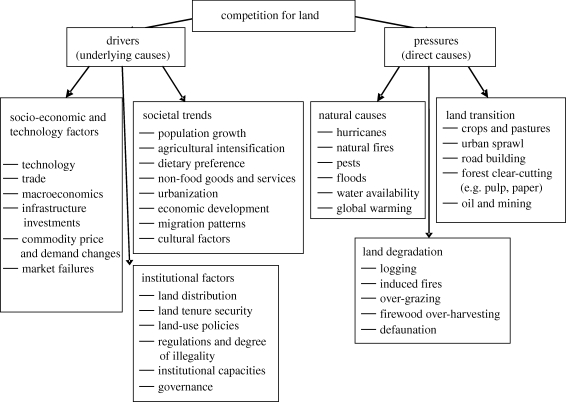
Conceptual analysis framework for competition for land, drivers and pressures. Adapted from Contreras-Hermosilla (2000).

Although competition for land has been identified as a driver affecting land use, food and farming by the Foresight Global Food and Farming Futures Project, it is actually an emergent property of a range of other drivers and pressures. Figure 1 presents a conceptual framework for analysing drivers and related pressures of competition for land at different geographical scales.

In understanding interrelated causes for competition for land, we distinguish between *drivers* and *pressures. Pressures* represent *direct causes*, the visible motivations for competition for land (right-hand side of figure 1). *Drivers* (*underlying causes*) for competition are factors of higher causal order that determine the degree of the actual direct pressures (left-hand side of figure 1), (see Chomitz & Gray 1996; Kaimowitz *et al*. 1998; Geist & Lambin 2002; Wunder 2003; Niesten *et al*. 2004; Rudel *et al*. 2005, on these different drivers and pressures; S. Klappa 1999, unpublished data).

We do not attempt to review the drivers and pressures in detail here, since they are covered by the other driver reviews in this issue. In §2*a*, however, we discuss a few drivers and pressures to demonstrate how they impact upon land use *through their impact on competition for land*.

### (a) Population growth, agricultural intensification and dietary preference

The growth in the human population from about 3 billion in 1960 to 6.8 billion in 2010, coupled with increased income and changes in diet, has been accompanied by substantial increases in crop and animal production (2.7-fold for cereals, 1.6-fold for roots and tubers and fourfold for meat; Foresight 2009). This increase will need to be maintained if the projected population of 9 billion by 2050 is to be sustained. Past increases in crop production have occurred as a result of both extensification (altering natural ecosystems to produce products) and intensification (producing more of the desired products per unit area of land already used for agriculture or forestry). Of the world's 13.4 billion ha land surface, about 3 billion ha is suitable for crop production (Bruinsma 2003) and about one-half of this is already cultivated (1.4 billion ha in 2008). The remaining, potentially cultivatable, land is currently beneath tropical forests, so it would be undesirable to convert this to agricultural land because of the effects on biodiversity conservation, greenhouse gas emissions, regional climate and hydrological changes, and because of the high costs of providing the requisite infrastructure. Therefore, increased yield and a higher cropping intensity will need to be the main driver behind future growth in food production (Bruinsma 2003). Table 1 shows that, according to the projection of Bruinsma, extensification will still contribute significantly to crop production in Sub-Saharan Africa (27%) and Latin America and the Caribbean (33%). There is almost no land available for expansion of agriculture in South and East Asia and the Near East/North Africa (and there may be loss of agricultural land to urban development) so that intensification is expected here to be the main means of increasing production (Gregory *et al*. 2002; Bruinsma 2003).

**Table 1.** Projected contributions (%) to increased crop production between 1997/99 and 2030. Adapted from Bruinsma (2003).

	land area expansion	increase in cropping intensity	yield increase
all developing countries	21	12	67
Sub-Saharan Africa	27	12	61
Near East/North Africa	13	19	68
Latin America and Caribbean	33	21	46
South Asia	6	13	81
East Asia	5	14	81

The main means to intensify crop production will be through increased yields per unit area together with a smaller contribution from an increased number of crops grown in a seasonal cycle. As cereal production (wheat, maize and rice) has increased from 877 million tonnes in 1961 to 2342 million tonnes in 2007, the world average cereal yield has increased from 1.35 t ha^−1^ in 1961 to 3.35 t ha^−1^ in 2007. Simultaneously, *per capita* arable land area has decreased from 0.415 ha in 1961 to 0.214 ha in 2007 (Foresight 2009). Put another way, had the increases in yield of the last 40–50 years not been achieved, almost three times more land would have been required to produce crops to sustain the present population; land that, as indicated above, does not exist unless unsuitable for cropping. Without changes in productivity, the growing population would have led to an even greater expansion in agricultural area than observed, and competition for land would have been greatly intensified.

There have also been substantial changes in human food consumption reflected in dietary and nutritional changes over recent decades (Schmidhuber 2003). There is an increasing demand for livestock products, particularly in developing countries (Smith *et al*. 2007), and given the lower efficiency of livestock products compared with the direct consumption of vegetal matter (Stehfest *et al*. 2009), an increasing proportion of livestock products in the diet is expected to increase competition for land.

### (b) Non-food goods and services

While agricultural production for food consumption is one of the predominant land-use activities across the globe, land is also used for the production of timber, fibre, energy and landscape amenities as well as being consumed by urbanization.

#### (i) Forest products and fibre

Historically, the production of forest products has grown rapidly—and again, in the future a further increase is necessary (upto 2030 by 1.4% per annum for sawnwood, and 3% for paper and wood-based panels; FAO 2009*a*). But worldwide, the area of forest and woodland has decreased over the past decade (FAO 2009*a*,*b*; Foresight 2009), mostly at the expense of agricultural expansion. However, regional differences in forest areas and timber production are stark, with declines occurring in developing countries, but forest expansion in developed countries (table 2).

**Table 2.** Changes in global forest areas as a function of country income groups. From World Bank (1994) as reported by Hannink (1997).

	current median rate of forest reduction	
World Bank income group	% yr^−1^	halving time (years)
low	−0.80	90
lower middle	−0.60	120
upper middle	−0.55	131
high	+0.20	360 (doubling time)
world	−0.60	120 years

The different trends between developed and developing countries arise from a number of factors that reflect competition with other land uses.

— *Wood substitutes*. Developed countries have replaced the use of wood as a source of fuel and in construction.
— *Agricultural expansion.* Demand for agricultural products has been growing only modestly in developed countries, but rapidly in developing countries.
— *Trade patterns.* Developing countries tend to export primary products.
— *Public services.* Forests have amenity value in developed countries, and subsequently are often protected from deforestation through policy.

While the area of forest in developed countries is increasing only slightly, demand for wood products has fuelled deforestation in other parts of the world. The global production of fibre crops has almost doubled between 1961 and 2007 (Foresight 2009), but the land area used to produce these crops has declined by about 10 per cent over the same period (FAOSTAT 2010). This reflects the increase in global consumption of fibre goods, but also the increased productivity (yields) of fibre crops. The area declines suggest that the competition between food and fibre production may be decreasing.

#### (ii) Energy crops

The growth of crops for bioenergy has been highlighted as a potential competitor for land with food crops. It is noteworthy, though, that the area occupied by bioenergy and its by-products in 2004 was only 14 Mha compared with 1500 Mha of crops (i.e. about 1% of the total cropped area) and 4500 Mha of pastures worldwide (IEA 2006). While the reasons for growing crops for bioenergy are complex, the use of land for them is likely to increase in the future (FAO 2009*b*). An important issue for competition for land is the potential clearing of new land for biomass crops. Using biomass for energy is likely to have both positive and negative competitive effects on food production and therefore on land, with national and regional policies beginning to reflect differing components of these inter-linkages. With global oil stocks becoming increasingly threatened (UKERC 2009), fossil fuel prices will inevitably continue to rise and alternative sources of energy will be needed, not least to maintain agricultural yields. Bioenergy is likely to fill a significant part of this emerging energy gap for agriculture, which in turn will require more integrated energy/agriculture/land-use policies to circumvent adverse impacts of competition for land.

#### (iii) Amenity activities and biological conservation

An increasing trend in some parts of the world is the use of land for amenity activities and/or biological conservation. This includes recreational uses such as public parks, golf courses and other sports facilities, as well as the conservation of traditional landscapes for their aesthetic, cultural or natural heritage value. Land competition between amenity and other uses depends strongly on geographical location, with stronger pressures for amenity use occurring on land near to urban centres. However, many cultural landscapes are multi-functional, being used, for example, for food or timber production, as well as offering amenity services. Setting aside land for amenity or conservation potentially increases competition for land on the remaining area, which we return to in §§4 and 5.

### (c) Land and soil degradation

Degradation of soil and land through inappropriate use or the addition of pollutants has been a topic of concern for many decades, because of the potential impact on biodiversity, and the availability of land for the human population to feed itself. Degradation of land intensifies competition for land, since it reduces the quantity of land suitable for a range of uses such as food production. ISRIC (1991) produced a world map of human-induced soil degradation based on the knowledge of 250 experts from six continents showing that of the 11.5 billion ha of vegetated land, 15 per cent was degraded. Erosion was the main process of degradation, and about 20 per cent of the agricultural land worldwide was moderately degraded and 6 per cent strongly degraded (Oldeman 1994). A more recent global assessment of land degradation (ISRIC 2008) identifies 24 per cent of land as degrading, mainly in Africa (south of the equator), SE Asia and southern China, North and Central Australia, the Pampas and parts of the boreal forest in Siberia and North America. Although cropland occupies only 12 per cent of land area, almost 20 per cent of the degrading land is cropland, with forests also over-represented (28% of area but 42% of degrading land). Some 16 per cent of the land area is improving, including cropland, rangeland and forests. Overall, the assessment shows the importance of natural catastrophic phenomena and human management in driving degradation, with the latter also instrumental in speeding up rehabilitation.

Agriculture almost always results in stresses being applied to land (for example, by reducing organic matter returns to soils or the imposition of a physical stress such as tillage), but the properties of some soils allow them to recover naturally and rapidly, while others may require amendments (e.g. inputs of fertilizer) or other physical interventions to regain their productive ability (Greenland & Szabolcs 1994). By reducing degradation rates or increasing rates of land rehabilitation, competition for land in areas containing degraded land could be reduced (Debeljak *et al*. 2009).

### (d) The role of policy

Agricultural policy in many developed countries is dominated by protectionism, established through trade tariffs and producer support (subsidies). Subsidies affect land-use decisions by influencing the types of land-use strategies adopted by a land manager. So, for example, farmers will only grow crops for which they receive financial support through direct payment. In this sense, subsidies tend to limit competition for land. Subsidies also distort markets on a global scale and influence the competitiveness of agricultural land use in other regions of the world. Conversely, policy liberalization often leads to land-use diversification as seen, for example, in New Zealand following the 1984 agricultural policy reforms (MacLeod & Moller 2006), which overnight led to the complete removal of production subsidies (Smith & Montgomery 2004). In doing so, however, a liberalized land-use policy is likely to increase competition between land uses.

Pressure from the World Trade Organization, among other drivers, has in part led the governments of the developed world to move away from production-related support to new policy directions based on rural development or environmental protection. Policies such as the Less Favoured Area scheme in Europe, for example, were designed with the objective of protecting agricultural land use in areas with a competitive disadvantage, usually because of physical limitations such as topography or climate. By preserving the *status quo* of traditional agricultural landscapes, such policies limit or remove entirely the competition between alternative land uses. Other policies such as the European agri-environment schemes compensate farmers for managing their land to high environmental protection standards. The common theme in rural development and environmental protection policies, however, is the support of farmer incomes, and this leads to the maintenance of current land-use practices that limits land competition.

Competition for land is associated with deforestation owing to agricultural expansion while, at the same time, expansion of forests is leading to competition with other land uses. Furthermore, permanent forest clearing is associated with the loss of many other ecosystem services. Thus, deforestation is not only a phenomenon of competition for land *per se*, but is also important in considering the wider concept of competition for ecosystem services.

## 3. Observed global trends in land use, 1990–2010

**Figure 2.**
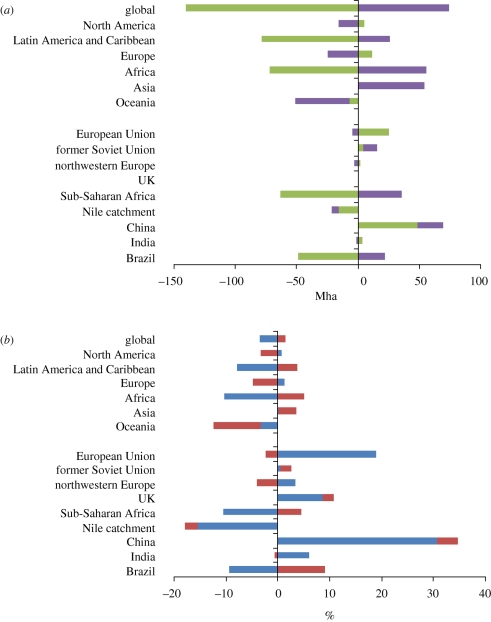
(*a*) Absolute and (*b*) percentage changes (of total agricultural and forest/wood area) in forest/wood and agricultural areas 1990–2007, globally and in different world regions. (*a*) Green bars, forest and wood (Mha); purple bars, agricultural land (Mha). (*b*) Blue bars, forest and wood (%); brown bars, agricultural land (%). Adapted from FAOSTAT (2010).

Since 1960, agricultural area has increased from just under 4.5 billion ha to just over 4.9 billion ha in 2007 (FAOSTAT 2010). During the last 20 years, there has been an overall increase in agricultural area from 4.86 billion ha in 1990, but showing some fluctuations, with the greatest area of 4.98 billion ha recorded in 2001. Figure 2 shows the absolute and percentage change in agricultural and forest/woodland area for the world, and for each world region, 1990–2007.

As described in §2, the close to tripling of global food production since 1960 has largely been met through increased food production per unit area. For example, Bruinsma (2003) suggests that 78 per cent of the increase in crop production between 1961 and 1999 was attributable to yield increases, and 22 per cent to expansion of harvested area. Land use has therefore changed, despite smaller changes in land cover.

While yield increases have outpaced increases in harvested area in most regions, the proportions vary. For example, 80 per cent of total output growth was derived from yield increases in South Asia, compared with only 34 per cent in Sub-Saharan Africa. In industrial countries, where the amount of cultivated land has

Box 1. **Models used for examining land-use change and competition for land in this review.**

The IMAGE Integrated Assessment Model (MNP 2006) is a modelling framework often applied in the field of land-use/land-cover change, because it is able to provide a geographically explicit description of land use. The land-use/land-cover description of IMAGE can be coupled to other models such as the global trade analysis project (GTAP) model or the IMPACT model. The rule-based allocation of IMAGE accounts for crop productivity and other suitability factors, such as proximity to existing agricultural land and water bodies. Bioenergy crops are grown on land other than that required for food production, forests, nature reserves and urban areas (MNP 2006).

The macro-economic EPPA model was developed to examine climate and energy policy applications. Future scenarios are driven by economic growth using the GTAP data as base information, simulating the economy recursively at 5-year intervals. Five land types are considered to be interchangeable without restrictions, as long as conversion costs are met (submodel EPPA-PCCR, Pure Conversion Cost Response; EPPA-PCCRN, normalized PCCR; and EPPA-OLSR, Observed Land Supply Response). Reversion to the natural state occurs under no costs, with any prior investment being fully depreciated.

The MiniCAM model is also an environmental integrated assessment model. Land is allocated between different categories according to its expected profitability. This is determined by the productivity of the respective product, product price, the rental rate of the land and the non-land cost of production. Managed and unmanaged ecosystems are interchangeable according to the above.

Quickscan is a spreadsheet-based model constructed to analyse the bioenergy potential under consideration of key drivers and correlations. The study considered here identified the consumption of animal products as a key factor for agriculture land use and examined alternative production systems. The resulting spared land is considered to be available for bioenergy production. Therefore, the study considered only had the land categories ‘bioenergy’ and ‘pasture’.

The GRAPE model is a macro-economic model of climate change. Land is allocated according to food demand of the population and the land-use rent that takes carbon costs (external costs caused by energy systems, land use and land-use change) into account.

For the MESSAGE-MACRO framework, integration of agriculture and forestry sectors has been achieved through linkages to the DIMA and AEZ-BLS models as described in Riahi *et al*. (2007). While land requirements for bioenergy supply and CO_2_ mitigation via forest-sink enhancement are based on the sensitivity analysis of the DIMA model, the AEZ-BLS framework provides inputs with respect to agricultural land expansion.

GLOBIOM integrates the agricultural, bioenergy and forestry sectors. Changes in the demand on the one side and profitability of the different land-based activities on the other side are the major determinants of land-use change. Spatially explicit land-use suitability and respective productivities, as well as environmental effects, are taken into account (Havlík *et al*. in press).

The IMPACT model projects global food supply, food demand and food security to the year 2020 and beyond. Demand is determined by prices, income and population growth. From cropland and urban land projections, only cropland is considered in this analysis.

been stable or declining, increased output was derived predominantly through the development and adoption of agricultural knowledge science and technology, which has served to increase yields and cropping intensity (van Vuuren *et al*. 2008). The role of land-use change and adoption of agricultural knowledge, science and technology have, therefore, varied greatly between regions. In some regions, particularly in Latin America, the abundance of land has slowed the introduction of new technologies (van Vuuren *et al*. 2008).

## 4. Projected global trends in land use, 2010–2050

### (a) Changes in land use

The previous sections have shown that land-use changes are a result of the interaction of a variety of drivers and pressures. In particular, population growth and a shift towards more meat-intensive diets have in the past contributed to an increasing demand for agricultural land. These factors are expected to continue to be important in the future, although trends will differ in time and across regions. Historically, the demand for more agricultural production has been partly compensated by technological advances, and improving technology will determine whether yields will continue to improve in the future.

The complexity of the interactions between different drivers necessitates the use of scenario studies using models of land resources and land use, to analyse the consequences of particular trends and policies. There is a variety of studies and a range of models for addressing these issues. Box 1 gives an overview of the most commonly used models for such analysis at the global scale. For a review of land-use change scenarios at the regional scale, see Alcamo *et al*. (2006); Busch (2006) and de Chazal and Rounsevell (2009).

Future land-use trends are described as part of studies that look into long-term agriculture trends (such as the projections published by the Food and Agriculture Organization of the United Nations (FAO) and International Food Production Research Institute (IFPRI). In addition, studies focusing on agricultural trade increasingly tend to describe the relationships between trade flows and land use. Finally, integrated assessment models, used for examining global environmental change and climate change, are increasingly applied to investigate how climate policies might interact with land-use change. The type of models used in these different areas vary greatly, ranging from models derived from the economic tradition (general equilibrium models, e.g. GTAP-type models) to partial agricultural-economy models (like IMPACT), and models that focus mostly on the interaction of economic activity and biophysical indicators (e.g. the IMAGE and GLOBIOM model; box 1). General equilibrium models account for the economic linkages of the land-use sector with the rest of the economy and allow for assessment of income generation owing to land-use activities. Another strength of these models is their consistent description of agriculture trade. Partial equilibrium models allow for detailed study of agricultural production of different crops and within different regions. Moreover, some of these models are also able to represent specific land-use-related policies. Biophysically based models allow the relationship between environmental parameters (production potential based on soils and climate; land cover), land use and agriculture to be studied. Within the scope of this paper, we will not be able to review the complete literature of land-use scenarios; instead we will focus on a few noteworthy projections (table 3), while in table 4 we provide some details on the selected models, emphasizing how these models handle land use. For full details, the reader is advised to consult the references given.

**Table 3.** Overview of studies considered in this review.

study	focus	model(s)	scenarios
IPCC-SRES (IMAGE)	providing different trajectories for global environmental change (especially climate change)	IMAGE	A1, B1, A2, B2
Millennium Ecosystem Assessment	providing contrasting futures with respect to the future of ecological services	IMAGE/IMPACT	Global Orchestration, Technogarden, Adapting Mosaic, Order from Strength
GEO-4	providing different trajectories for global environmental problems	IMAGE/IMPACT	Markets First, Policy First, Security First, Sustainability First
IAASTD	describing alternative future for agriculture with focus on the role of agricultural technology and knowledge	IMAGE/IMPACT	reference scenario and variants
FAO projections	exploring most likely developments for agriculture	IMAGE	reference scenarios in subsequent studies
Stehfest	exploring impact of different consumption behaviour on land use	IMAGE	healthy diet
IFPRI projections	exploring most likely development for agriculture	IMPACT	—
MIT studies	exploring land-use implications of a global biofuel industry	EPPA-PCCRN	ref/policy
		PCCR	
		OLSR	
Wise *et al*. (2009*b*); Gillingham *et al*. (2008)	exploring relationships between climate policy and land use	MiniCAM	
Smeets *et al*. (2007)	exploring potential for bioenergy	Quickscan	
Kosugi *et al*. (2009)	exploring the effect of internalization of external costs into the model on land-use results	GRAPE	
IIASA Greenhouse Gas Initiative Scenarios	providing different trajectories for global environmental change with focus on climate mitigation	BLS/DIMA/MESSAGE	A2r, B2, B1
Havlík *et al*. (in press)	exploring relationship between bioenergy, climate policy and land use	GLOBIOM	updated baseline

**Table 4.** Overview of models considered in this study.

tool	type	developed at	reference
IMAGE (Integrated Model to Assess the Global Environment)	integrated assessment model	National Institute for Public Health and the Environment (RIVM) and the Netherlands Environmental Assessment Agency (MNP)	MNP (2006)
EPPA (Emission Prediction and Policy Analysis)	recursive-dynamic multi-regional computable general equilibrium model	MIT Joint Program on the Science and Policy of Global Change	Gurgel *et al*. (2007)
MiniCAM	integrated assessment model	Joint Global Research Institute	Wise *et al*. (2009*a*,*b*) and Gillingham *et al*. (2008)
Quickscan	bottom-up Excel spreadsheet model	Corpernicus Institute for Sustainable Development and Innovation	Smeets *et al*. (2007)
GRAPE (Global Relationship Assessment to Protect Environment)	integrated (bottom-up) model to assess the global environment	Japan	Kosugi *et al*. (2009)
MESSAGE (Model for Energy Supply Strategy Alternatives and their General Environmental impact)	integrated assessment modelling framework	International Institute for Applied Systems Analysis (IIASA)	Riahi *et al*. (2007)
GLOBIOM (Global Biomass Optimization Model)	recursive dynamic multi-regional partial equilibrium bottom-up model for agriculture, forestry and bioenergy	International Institute for Applied Systems Analysis (IIASA)	Havlík *et al*. (in press)
IMPACT (International Model for Policy Analysis of Agricultural Commodities and Trade)	partial-equilibrium agricultural model for crop and livestock commodities	International Food Policy Research Institute (IFPRI)	Rosegrant *et al*. (2001, 2008)

#### (i) Studies on land use

The most widely used agricultural projections are those of FAO and IFPRI. IFPRI uses the IMPACT model as the basis of its projections. The methods underlying the FAO projections are more diverse, utilizing both models and expert consultations. Both studies consider mostly agricultural markets, and thus do not fully cover land-use projections. The scenario projections in the Global Environmental Outlook-4 (UNEP 2007), the Millennium Ecosystem Assessment (MEA 2005) and the IIAASTD study (van Vuuren *et al*. 2008) all focused on the relationship between environmental change and the agriculture sector. In these studies, a combination of the IMAGE model and IFPRI's IMPACT model was used to define the scenarios. The scenarios of the other studies look at more specific cases in regard to climate policy and biofuel potential. The general trends common to the scenarios considered here show an increase in land for bioenergy, crops and livestock, with forest and other lands decreasing. The exceptions here are scenarios implementing a carbon tax and a lower meat diet where more land is converted back to unmanaged forest. The scenarios considered by the IMAGE model, and those used in a wider range of studies, are given in tables 5 and 6, respectively. Table 7 shows the different land categories considered by each of the models we compare in this section.

**Table 5.** Scenario descriptions of studies using IMAGE derivations.

scenario abbreviation as used in figures	description
SRES	
A1	high economic growth and rate of innovations, environmental issues get addressed
A2	self-reliance and preservation of local identities
B1	assumes continuing globalization and economic growth, and a focus on the environmental and social—immaterial—aspects of life
B2	local solutions to economic, social and environmental sustainability
MA (Millennium Assessment)	
GO (Global Orchestration)	globalized with emphasis on economic growth
OS (Order from Strength)	regionalized with emphasis on security
TG (Technogarden)	globalized with emphasis on green technology
AM (Adapting Mosaic)	regionalized with emphasis on local adaptation and flexible governance
GEO4	
MF (Markets First)	focus on markets, not only to deliver economic advances but also social and environmental improvements
SecF (Security First)	focus on security issues, in a strongly regionalized world
SusF (Sustainability First)	focus on sustainability issues, integrating environmental and social concerns at the heart of development decisions at every level of scale
PF (Policy First)	focus on global, coordinated corrections to the ‘Market First’ scenario without changing the underlying paradigm emphasizing economic growth
OECD EO	
baseline	no new policies
450 ppm	stabilization of greenhouse gas to 450 ppm by 2100
IAASTD	
baseline	slowly declining rates of growth in agricultural research
high AKST	higher crop yield and livestock number growth
IMAGE-FAO	
reference	reference meat diet
Stehfest *et al*. (2009)	
healthy diet	‘healthy eating’ recommendations implemented globally (reducing meat consumption and increasing consumption of vegetables)

**Table 6.** Descriptions of all scenarios considered in this review, not included already in table 5.

scenario abbreviation as used in figures	description
EPPA-PCCR, -PCCRN and -OLSR	
ref/BAU	business as usual with no attempt to control greenhouse gas
policy	global effort to control greenhouse gas emissions starting with the Kyoto protocol—reflects a path whereby developed countries would reduce emissions by 50% by the year 2050
MiniCAM: Wise *et al*. (2009*a*)	
ref	future estimates of crop productivity are applied to terrestrial products until 2030; then a rate of 0.25% per year
FFICT	Fossil Fuel and Industrial Emissions Carbon Tax regime
UCT	Universal Carbon Tax regime
Gillingham *et al*. (2008)	
B2	implements SRES B2 scenario
B2_550	as above with implementation of a mitigation policy to achieve atmospheric CO_2_ of 550 ppmv by 2095
Quickscan: Smeets *et al*. (2007)	
system 1	mixed animal production, rainfed agriculture
system 2	mixed animal production, rainfed and irrigated agriculture
system 3	landless animal production, rainfed and irrigated agriculture
system 4	landless animal production, very high crop production technology, rainfed and irrigated agriculture
GRAPE: Kosugi *et al*. (2009)	
GRAPE (B2)	economic cost of environmental impact in a case of successful internalization of externalities
MESSAGE: Riahi *et al*. (2007)	
A2r	based on SRES A2 with a lower population growth
GLOBIOM: Havlík *et al*. (in press)	
updated baseline	the published baseline was updated in several aspects, where the major ones are: macro-economic drivers and bioenergy projections from POLES scenario for Copenhagen communication. Introduction of bioenergy poly-production, higher land-use change flexibility including cropland expansion to grassland and other natural land and non-zero exogenous input neutral crop productivity growth (0.5% p.a.)

**Table 7.** Comparison of land categories used in different models. Land categories in *italics* are used in figure 6. Plus symbol, 100% match with used land category.

land category	IMAGE	EPPA	MiniCAM	GRAPE	GLOBIOM	MESSAGE
*bioenergy*	+	+	+	n.a.	n.a.^b^	n.a.
agricultural land						
*cropland*	+	+	wheat, corn, fibre crop, misc. crop, oil crop, other grain, sugar crop, rice, other arable land	+	+	+
*pasture*	grass and fodder crop	+	pasture and fodder crop, unmanaged pasture	grassland	grassland	intensive grazing, pasture
*forest*						
managed forest	regrowth forest (timber)	+	+	only total forest	plantation forest, managed forest	only total forest
unmanaged forest	Regrowth forest (abandoning), wooded tundra, boreal forest, cool conifer, temperate mixed and deciduous forest, warm mixed, tropical woodland, tropical forest	+	+	only total forest	+	only total forest
*other*	grassland/steppe, scrubland, savannah	natural grassland	grassland and shrubland	within other land	other natural vegetation	extensive grazing, non-vegetated land
included in respective model but not considered in this review	desert, ice, tundra	tundra, wetlands, desert and built-up areas are not explicitly represented in the model	desert^a^, urban^a^, tundra^a^	urban		build-up land, water

^a^Only in Wise *et al*. (2009*b*).

^b^Bioenergy is included within plantation forest and cropland.

#### (ii) Changes in consumption

**Figure 3.**
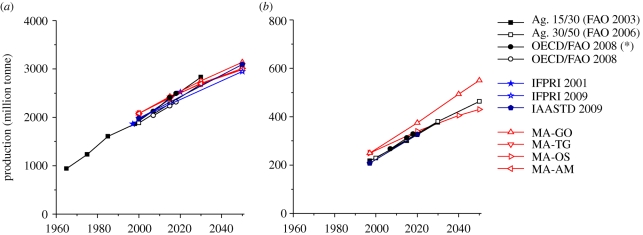
Trend in global production of (*a*) cereals and (*b*) meat according to various assessments. MA scenarios are from Carpenter & Pingali (2005); the OECD/FAO study has been included with (asterisk) and without biofuels; IFPRI 2009 is reported by Msangi & Rosengrant (2009).

Global food production is projected to increase, driven by population growth and changes in diet (§2*a*). The increase in production is somewhat slower than in the past, as a result of a slowdown in population growth. Diets are projected to become more meat-intensive, with annual *per capita* meat consumption increasing. The growth in production of cereals over the 2000–2050 period, based on a range of assessments, varies between 43 and 60 per cent (figure 3). The differences are relatively small since estimates of consumption growth are mostly driven by the increase in the global population (which shows relatively little variation between the different scenarios in 2050). An increasing share of cereals will be used as animal feed to supply the rapidly growing demand for livestock products. As incomes increase, demand for animal products also increases. This trend, which has been empirically established in all regions, is assumed to continue in the scenarios of the three groups of studies considered here. As a result, meat demand is projected to increase at a greater rate than the global population, and diets are projected to become more meat-intensive. For instance, the IFPRI calculations show annual *per capita* meat consumption increasing, on average, from 90 kg per person per year to over 100 kg between 2000 and 2050 in high-income countries, and from around 25 kg to nearly 45 kg per person per year in low-income countries during the same period. This trend is relevant for land use, since animal products require much more land than crops. On average, the production of beef protein requires several times more amount of land than does the production of vegetable proteins, such as cereals (Stehfest *et al*. 2009). While meat currently represents only 15 per cent of the total global human diet, approximately 80 per cent of the agricultural land is used for animal grazing or the production of feed and fodder for animals (FAO 2006). It should be noted that this includes extensive grasslands in areas where other forms of agriculture would be extremely challenging. Interestingly, future meat production varies considerably more than future cereal production among the scenarios (figure 3), since different scenarios show much more divergence in *per capita* meat consumption than for *per capita* cereal consumption. Some studies have looked into the consequences of reducing consumption of livestock products, with proteins being substituted by additional consumption of pulses (Stehfest *et al*. 2009), and shown that far less land would be required for agriculture under such extreme scenarios.

#### (iii) Cropland

**Figure 4.**
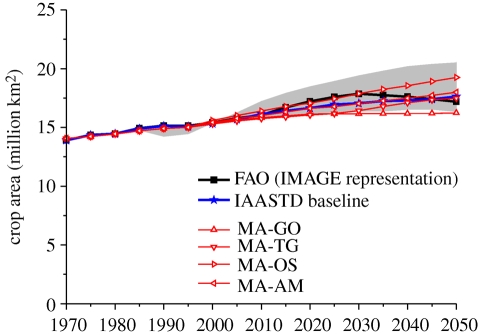
Change in crop area in various assessments (IAASTD projection includes land for bioenergy crops). Grey area indicates 20–80th percentile literature range.

**Figure 5.**
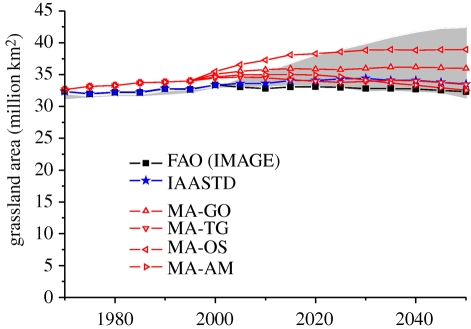
Projected change in grazing area in various assessments. Grey area indicates 20–80th percentile literature range.

**Figure 6.**
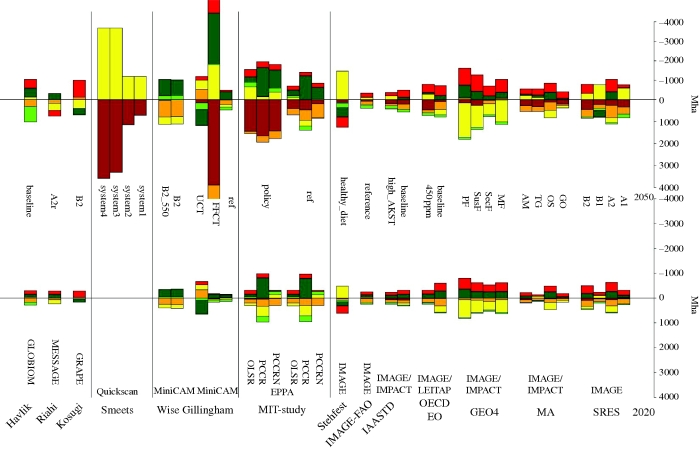
Global land-use change by 2020 and 2050 for different models and scenarios (see tables 5 and 6 for abbreviations). Change given as absolute change relative to 2000 with the exception of MiniCAM (base year 2005) and GRAPE (base year 2010) where this was the nearest available year. Table 7 details the land categories for the different models. Brown, biofuel; orange, crop; yellow, pasture; light green, managed forest; dark green, unmanaged forest; red, other.

The actual demand for cropland in the future depends on the balance between increases in agricultural demand and increases in yield improvement. Historically, yield improvements (approx. 80%) have been more important in increasing production than expansion of agricultural land (approx. 20%; see §§2 and 3 for more details). As a result, agricultural areas have expanded by about 5 per cent since 1970. Scenarios show a very large variation in the expected development in cropland (figures 4–6). The 2050 projections for cropland increase range from as low as 6 per cent (e.g. the Technogarden scenario of the MEA), to an increase of more than 30 per cent (such as for the SRES A2 scenario, and one of the scenarios of the EPPA model; numbers represent the 60% interval of the literature). The average increase is around 10–20% (see also van Vuuren *et al*. 2008). In general, models with a stronger focus on physical parameters tend to project somewhat lower growth rates than models with a more macro-economic orientation (figure 6).

**Figure 7.**
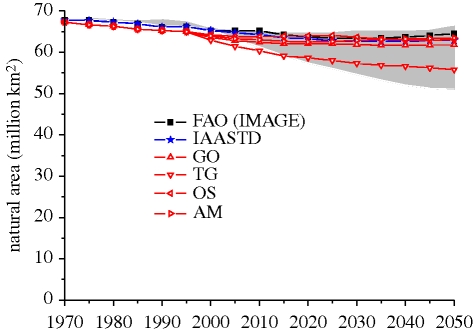
Remaining natural area according to projections from various assessments (deserts and ice areas are not included). Grey area indicates 20–80th percentile literature range.

The slightly lower contribution (on average) from the expansion of crop area can be attributed to increasing land scarcity and reduced growth of the global population. The decreasing quality of land brought into production, however, may mean that a greater percentage of gains in total production will need to be found from crop area expansion than has historically been the case (as indicated in MEA 2005). Even in the two scenarios with little global expansion of cropland, a considerable expansion of arable land still occurs in Africa, Latin America and partly in Asia, but this is compensated for by a decrease in arable area in temperate zones. Across the assessments, the area in crop production increases from 1.4 billion ha (or 10% of Earth's land surface) to up to 2.3 billion ha. As indicated by FAO, this expansion is within the scope of total land available for crop production (Bruinsma 2003). The fact that the assessments considered here agree on a rather flexible continuous response of the agricultural system to demand increases is interesting, as more sceptical views have also been expressed. An important implication, however, is further loss of the area available for unmanaged ecosystems (figures 4 and 7).

#### (iv) Animal husbandry and pastures

Increases in meat production will occur through a number of means, including changes that lead to intensified production systems, such as more efficient conversion of feed into animal products, and via expansion of land use for livestock (figure 6). Previous scenarios indicate that most of the increases in world livestock production will occur in developing countries (Bouwman *et al*. 2005). For grazing systems, this means that some intensification is likely to occur. Considerable intensification is likely in mixed systems, with further integration of crop and livestock in many places. Strong growth is implied for confined livestock production systems. In the FAO scenario, for instance, at least 75 per cent of the total growth is in confined systems, although there are likely to be strong regional differences (e.g. less growth of these systems in Africa; Bruinsma 2003). This is a continuation of historic trends. The major expansion in industrial systems has been in the production of pigs and poultry, as they have short reproductive cycles and are more efficient than ruminants in converting feed concentrates (cereals) into meat. Industrial enterprises now account for 74 per cent of the world's total poultry production, 50 per cent of pig meat and 68 per cent of eggs (FAO 2006, 2009*a*,*b*). At the same time, a trend to more confined systems for cattle has been observed, with a consequent rapid increase in demand for cereal- and soy-based animal feeds (these trends are included in the projections discussed in the previous section; see Delgado *et al*. 1999).

For grazing land, the range of 2050 scenario projections ranges between a 5 per cent contraction to a 25 per cent increase (60% interval). Most studies show an increase of 10 per cent or less. The IAASTD baseline, for instance, projects an almost constant grazing area (van Vuuren *et al*. 2008). These numbers are lower than for croplands, representing the general view that croplands are expected to grow faster than the grazing area, driven by a further intensification of livestock production systems (and despite the rapid growth in meat consumption). The vast area of land used for animal husbandry also means that some studies looking into alternative pathways for land use often identify a large potential for reduction here, either by low-meat diets (Stehfest *et al*. 2009), or intensification (Smeets *et al*. 2007).

#### (v) Total land balance

Obviously, the total demand for agricultural area arises from trends in cropland and grassland. Studies show diverging trends (figure 6), but there are also some common characteristics. First of all, almost all studies show an expansion in 2020 and 2050 of the area for cropland and grassland (as already noted in the previous sections). Second, in most studies, expansion of grassland area or cropland area represents the most dominant expansion category in 2020; by 2050, in some studies, however, bioenergy also becomes important (especially EPPA, MiniCAM and Quickscan). As indicated earlier, cropland expansion is generally more important than expansion of grassland, but there are some noteworthy exceptions (GEO4, and EPPA in 2020). In nearly all studies, both forest area and other areas (savannah, natural grasslands etc.) decline. The lowest numbers of land-use change are reported for the MEA (2005) scenarios, the IAASTD scenario, the IMAGE representation of the FAO baseline and the MiniCAM reference. Some of these scenarios include high levels of technology change (Global Orchestration, Technogarden and high AKST). High rates of land-use change are reported for several of the EPPA and MiniCAM scenarios. It should be noted that figure 6 represents a global picture. Much more change may happen at the regional level. A considerable expansion of arable land still occurs in Africa, Latin America and partly in Asia, but this is compensated for by a decrease in harvested area in temperate zones.

An important implication, however, is further loss of the area available for unmanaged ecosystems. This is already shown in figure 6; figure 7 shows the remaining natural areas globally—but again it should be noted that these global figures hide underlying regional trends. In general, across the assessments, total natural areas decline by about 0–20%. This includes so-called baseline projections; but also scenarios that focus more on the projection of ecosystem services such as the MEA's Technogarden scenario; or the Sustainability First scenario of GEO4. There are only a few studies that have looked at incremental switches in management systems, such as those to semi-natural forest management (e.g. Havlík *et al*. in press) and changes in grassland management. A great impact on land-use change can also come from carbon incentives as demonstrated by Wise *et al*. (2009*a*). For example, the scenario examined by Wise *et al*. (2009*a*) in which (i) it is assumed that greenhouse gas emissions of the energy system are regulated and (ii) there is no regulation of emission from land-use change, according to MiniCAM work, will result in massive land-use change towards bioenergy and crops. In contrast, a policy that targets all potential greenhouse gas emissions (also from land use) can lead to preservation of woodland. A similar trend can be observed from the GRAPE model, which also takes carbon cost into account. In fact, these studies suggest that carbon taxing could have an impact on changing diet via the induced prices of meat.

Ever-increasing competition for land may endanger the integrity of currently protected areas, which are located and classified in the World Database on Protected Areas (UNEP-WCMC 2009). Most model studies discussed above either assume projected areas to be constant, or even ignore this category as a special land category. There is one major exception, which is the Sustainability First scenario as part of UNEP's GEO4. Based on a minimum share of protected land by biome category, this study assumes that projected area would need to increase from 2009 to 2030 by up to approximately 400 Mha worldwide. Many of these areas may not enter into strong competition with other land uses, while some are clearly at the forest frontier.

## 5. Uncertainty

Uncertainties in projecting land use have a range of sources, including the level of understanding of the underlying causal relationships (i.e. ‘what is known about driving forces, their impacts and interdependencies?’), the degree of complexity of underpinning system's dynamics (i.e. ‘how do driving forces, impacts and their respective feedbacks lead to emerging nonlinear system dynamics?’), the degree of path dependency (i.e. ‘to what degree does the current system state and past trends determine future developments?’), the level of uncertainty introduced by the time horizon (i.e. ‘how far into the future?’) or even surprises and unpredictable future developments. Some of these phenomena follow known random processes while others cannot be explored well enough as we lack anticipative capacity. For a more complete discussion of different types of uncertainties and their consequences for methods to explore the future, see van Vuuren (2007).

This section serves to illustrate some of these uncertainties inherent in future projections of land use and of competition for land, and how these are critically dependent upon future policies on forest protection and bioenergy supply, and future trends in agricultural product preferences and consumption.

Given that there is substantial uncertainty about how different drivers will evolve and how they will impact upon the competition for land, here we illustrate the impact of uncertainty by presenting results of eight selected changes of drivers between 2020 and 2030. The analysis presented here was carried out using the GLOBIOM model (table 4 and box 1; Havlík *et al*. in press) over a short timeframe, to reduce the level of uncertainty introduced by the time period considered. Four uncertainty domains were identified for the quantitative modelling analysis on a global scale: biofuel, meat and wood demand and infrastructure development. In total, eight alternative scenarios were modelled under these four uncertainty domains, since the biofuel scenarios included five variants, differentiated by the expected biofuel mix (table 8). To assess uncertainty in this analysis, the policy shock (table 8) was incorporated in the baseline for each scenario separately, and the model was re-run with the new assumptions.

**Table 8.** Policy shock scenarios used in the GLOBIOM model analysis.

scenario name	description
baseline	POLES scenario for Copenhagen communication: 8.3% of biofuel in total transport energy in 2030
biofuels—portfolio	BIOF1 = 15% share of biofuels in total transport energy in 2030 in the form of a mix of all three types of biofuels (first-generation biodiesel and ethanol and second-generation bioethanol)
biofuels—ethanol	BIOF2 = 15% share of biofuels in total transport energy in the form of first-generation ethanol only in 2030
biofuels—biodiesel	BIOF3 = 15% share of biofuels in total transport energy in the form of first-generation biodiesel only in 2030
biofuels—first generation	BIOF4 = 15% share of biofuels in total transport energy in 2030 from first generation (mix of biodiesel and bioethanol) only
biofuels—second generation	BIOF5 = 15% share of biofuels in total transport energy in 2030 from second generation only.
wood	WOOD = overall additional increase of 15% in demand for wood in 2030
meat	MEAT = overall additional increase of 10% for meat in 2020 and 15% in 2030
infrastructure	INFRA = transportation costs will decrease by 10% in emerging economies and 5% in developing regions by 2030

The scenarios were defined in such a way that any expansion of cropland would occur at the cost of forest land in order to have a ‘pure’ measure of the degree of competition for land. Under this scenario specification, the GLOBIOM model considers only drivers of deforestation coming from agriculture or bioenergy production. We consider that the model operates under the constraint of a fixed total land area, and allocates land use according to the economic competitiveness of different land-use activities. Deforestation is used as a measure of the degree of competition for land and is itself costly. The cost of avoiding deforestation is equal to the difference between the cost of deforestation itself and the income from agricultural production that would occur on that land subsequently if it were deforested and used for agriculture (opportunity cost). Under avoided deforestation, the degree of competition for land is mitigated at the cost of land-use intensification and reduced consumption (Havlík *et al*. in press).

**Figure 8.**
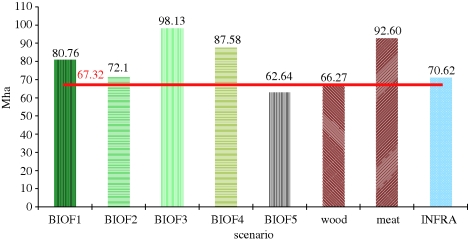
Global deforested area owing to expansion of agricultural land between 2020 and 2030 (Mha). Red line, baseline.

Figure 8 presents the global deforested area, which serves as a proxy for the degree of competition for land, between 2020 and 2030 in Mha. The red line displays the baseline scenario. The biofuel scenarios 1, 3 and 4, and the meat policy shock scenario, cause more deforestation. These scenarios are associated with agricultural land expansion owing to additional production of commodities. Improvement of infrastructure in emerging and developing economies on the one hand leads to higher pressure on natural ecosystems on the frontier, and on the other hand increases global productivity of agricultural production, and will therefore reduce land expansion in the long term. The infrastructure scenario leads to some 3 Mha more deforestation compared with the baseline. The result for the wood scenario is very close to baseline results, causing 0.35 Mha less for additional wood consumption since the relative value of forest increases. The only scenario that leads to less deforestation is the fifth biofuel scenario in which second-generation biofuels are used. This is associated with afforestation activities using high-yielding short-rotation forests. This policy shock scenario leads to a reduction in deforestation of more than 5 Mha over the period 2020–2030, when compared with the baseline.

The scenarios demonstrate the range of impacts a single biofuel production policy shock can exert on deforestation depending on the type of biofuel production technology used. Further sources of uncertainty lie in the resolution and quality of the land category considered. Many studies do not distinguish between managed and unmanaged forest and do not consider conversion to short-rotation coppice as deforestation. Therefore, in terms of net deforestation, natural forest can be converted in such models to short-rotation coppice without showing land-use change. In the scenarios presented here, deforestation is defined as conversion of unmanaged natural forest to cropland. The development of different forest types was tracked separately. For example, short-rotation plantations were only allowed to expand into cropland and grassland and therefore could only indirectly lead to deforestation through cropland expansion elsewhere into unmanaged forest. Increasing forest management intensity does not lead to deforestation. Lower deforestation in the second-generation biofuel and WOOD scenario is due to the increased value of managed forest, reducing deforestation as described above. However, the increased value of forest management leads to higher conversion of unmanaged forest to managed forests using semi-natural forest management practices. Another source of uncertainty arises from the models themselves. All models provide an imperfect representation of reality and rely on the availability and quality of input data and additional assumptions. For example, in GLOBIOM, there is no explicit link assumed between increased animal production and grassland demand. Consequently, the MEAT scenario will overestimate the degree of deforestation owing to the restrictive grassland assumptions. It is important to be aware of these inherent uncertainties when dealing with future projections. Improved models, data and more sophisticated scenarios will allow this uncertainty to be reduced in the future, but projections of future policy impact will always contain a degree of uncertainty.

## 6. Concluding remarks

We have shown that competition for land, in itself, is not a driver affecting food and farming in the future, but is an emergent property of other drivers and pressures. There is considerable uncertainty over projections of intensity of competition for land in the future, and the regional distribution of this competition. Modelling studies show that future policy decisions in the agriculture, forestry, energy and conservation sectors could have profound effects, with different demands for land to supply multiple ecosystem services usually intensifying competition for land in the future.

Given the need to feed 9 billion people by the middle of this century, and increasing competition for land to deliver non-food ecosystem services, it is clear that per-area agricultural productivity needs to be maintained where it is already close to optimal, or increased in the large proportion of the world where it is suboptimal. It remains a challenge to deliver these increased levels of production in a way that does not damage the environment and compromise other ecosystem services (Royal Society 2009).

In summary, in addition to policies addressing agriculture and food production, further policies addressing the primary drivers of competition for land (population growth, dietary preference, protected areas, forest policy) could have significant impacts in reducing competition for land. Technologies for increasing per-area productivity of agricultural land will also be necessary. Key uncertainties in our projections of competition for land in the future relate predominantly to uncertainties in the drivers and pressures within the scenarios, uncertainties in the models and data used in the projections and the policy interventions assumed to affect the drivers and pressures in the future. Though price has been used as an indicator of land scarcity and, therefore, competition for land, the development of other indicators to assess the intensity for competition for land is in its infancy, and the development of new metrics will advance our understanding of competition for land in the future.
